# Complicated Appendicitis in Pregnancy Masquerading as Septic Abortion: A Case Report

**DOI:** 10.7759/cureus.91452

**Published:** 2025-09-02

**Authors:** Sweta Singh, Girija S Mohanty, Tanmaya Maharana

**Affiliations:** 1 Obstetrics and Gynecology, All India Institute of Medical Sciences, Bhubaneswar, Bhubaneswar, IND; 2 Obstetrics and Gynecology, Postgraduate Institute of Medical Education and Research (PGIMER), Chandigarh, IND; 3 General Surgery, All India Institute of Medical Sciences, Bhubaneswar, Bhubaneswar, IND

**Keywords:** acute complicated appendicitis, appendicitis in pregnancy, diagnosis of appendicitis in pregnancy, emergency appendectomy, management of appendicitis in pregnancy

## Abstract

The early diagnosis of complicated appendicitis in pregnancy remains challenging. We report the case of a 25-year-old multiparous woman with complicated appendicitis masquerading as septic abortion in the first trimester. She was referred to us on the second post-abortal day with persistent abdominal pain. Two days earlier, at 12 weeks of gestation, she had presented to her local hospital with lower abdominal pain and vaginal bleeding. She was diagnosed with inevitable abortion, and a suction and evacuation procedure was performed. On presentation to our center, she was septicemic with features of systemic inflammatory response syndrome. She was febrile, with a blood pressure of 100/70 mm Hg, a total leukocyte count of 14,210/cumm, and 79.1% neutrophils. Ultrasound revealed a bulky uterus with an intact contour and a septated pelvic collection. A provisional diagnosis of septic abortion was made, and she was started on broad-spectrum antibiotics. After 24 hours, her condition showed no improvement. Contrast-enhanced CT revealed multiple fluid collections. An emergency laparotomy, following a failed laparoscopic attempt, demonstrated a perforation at the base of the appendix along with a fecalith in the peritoneal cavity. She was discharged on postoperative day 7 and was doing well at follow-up. Histopathology confirmed appendicular perforation, which probably caused the first-trimester abortion. A high index of suspicion, combined with imaging modalities such as ultrasonography and/or MRI/CT, is essential for the early diagnosis of complicated appendicitis in pregnancy. Early surgical intervention in cases of appendicular perforation complicating pregnancy is crucial to achieve optimal outcomes.

## Introduction

Appendicitis is the most common cause of acute abdomen worldwide [1]. Its estimated incidence is one in 1000 persons per year, with the lifetime risk being slightly higher in men (8.6%) than in women (6.7%) [2]. Acute appendicitis may be uncomplicated or complicated. Uncomplicated appendicitis refers to inflammation of the appendix without signs of necrosis or perforation, while complicated appendicitis is characterized by transmural or focal necrosis, which may lead to perforation [3]. The incidence of acute appendicitis during pregnancy is approximately one in 1000 [4].

Distinguishing between complicated and uncomplicated appendicitis is crucial, as treatment and prognosis differ. Delayed diagnosis and treatment in pregnant women with complicated appendicitis significantly increase the risk of maternal complications and fetal loss [4]. Each day of delay to surgery has been associated with a 23% increase in the odds of preterm delivery, preterm labor, or abortion [5]. Composite 30-day postoperative morbidity was reported to be nonsignificantly higher in pregnant women than in nonpregnant women (3.9% vs. 3.1%), with the exception of pneumonia, which occurred more frequently in pregnant women (0.7% vs. 0.2%) [6].

Various scoring models have been developed in the nonpregnant population to aid in the early diagnosis of complicated appendicitis [7]. The first nomogram for predicting complicated appendicitis in pregnancy was developed only recently [4]. This nomogram is significant, as it uses a minimal number of clinical variables (gestational age, CRP, and neutrophil percentage) to provide a prediction model for complicated appendicitis in pregnancy, enabling optimal treatment choices.

Against this backdrop, we report the case of a 25-year-old multiparous woman with perforated appendicitis masquerading as septic abortion and discuss the diagnostic and therapeutic challenges.

## Case presentation

A 25-year-old multiparous woman, on the second day following suction evacuation for inevitable abortion, was referred to us due to persistent abdominal pain. She had presented two days earlier to her local hospital with lower abdominal pain and vaginal bleeding in early pregnancy. She had been diagnosed with an inevitable abortion, and a suction evacuation procedure had been performed. However, as her abdominal pain did not subside, she was referred to a higher tertiary care center.

Upon presentation, her history was reviewed. She was a third gravida with two previous full-term normal deliveries who had presented to her local hospital at 12 weeks of gestation with lower abdominal pain of moderate intensity and mild vaginal bleeding. There was no dysuria or radiation of pain, and her bladder and bowel habits were normal. On general examination, she was conscious, oriented, and febrile. Her pulse rate was 94 beats per minute, blood pressure was 100/70 mm Hg, temperature was 39°C, and respiratory rate was 14 breaths per minute. Auscultation of the chest revealed bilateral basal crepitations, probably due to systemic inflammation.

**Figure 1:**
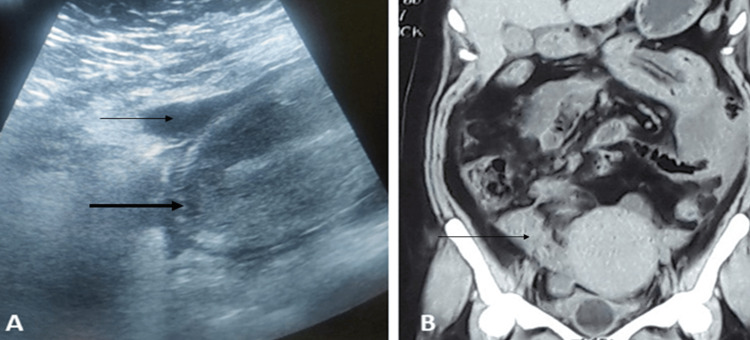
Preoperative imaging (A) Gray-scale ultrasound showing a mildly bulky uterus with an intact uterine contour (thick arrow) and the presence of ascites (thin arrow). (B) Contrast-enhanced CT of the abdomen showing multiple pockets of fluid collection (arrow) in the pelvis, suggestive of peritonitis.

On per-abdominal examination, tenderness was present in the lower abdomen, and bowel sounds were sluggish. Per speculum examination revealed a normal cervix with mild bleeding per vaginum. On bimanual examination, the uterus was bulky, soft, and mobile, with bilateral forniceal tenderness. Laboratory investigations showed a hemoglobin of 11.7 g/dl, a total leukocyte count of 14,210/cumm, neutrophils of 79.1%, and a CRP of 33.8 mg/L. Liver and kidney function tests were within normal limits. Gray-scale ultrasound showed a bulky uterus with no breach in the uterine contour and a septated pelvic collection (Figure 1A).

A provisional diagnosis of septic abortion was made. Differential diagnoses of surgical conditions such as appendicitis were also considered but deemed less likely. She was resuscitated, and broad-spectrum intravenous antibiotic therapy was initiated with ceftriaxone, gentamicin, and metronidazole, following the medical management protocol for septic abortion.

After 24 hours, there was still no symptomatic improvement in her condition. She had persistent lower abdominal pain of moderate intensity, and tenderness was elicited, although there was no rigidity or abdominal guarding. Her pulse rate was 90 beats per minute, blood pressure was 100/60 mm Hg, temperature was 39°C, and respiratory rate was 14 breaths per minute. Laboratory evaluation showed a total leukocyte count of 14,000/cumm with 80.0% neutrophils. She was advised to undergo an MRI of the pelvis; however, due to cost constraints, she declined. In the interest of time, contrast-enhanced CT was performed, which revealed multiple pockets of fluid collection (Figure 1B) with thick, enhancing peritoneum predominantly in the pelvis, suggestive of peritonitis.

**Figure 2:**
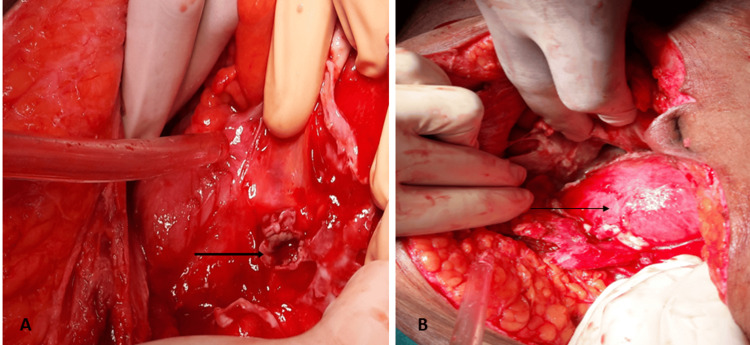
Intraoperative image (A) Site of rupture at the base of the appendix (arrow). (B) Intact uterine fundal contour (arrow).

In view of suspected peritonitis in the setting of septic abortion, she was planned for diagnostic laparoscopy. However, due to dense adhesions between the omentum and bowel loops, the procedure was converted to an open technique. On laparotomy, a purulent collection was found in the pouch of Douglas, inter-bowel pockets, and the subhepatic space, along with omental adhesions and pelvic inflammation. Exploration of the bowels revealed a perforation at the base of the appendix (Figure 2A) along with a fecalith in the peritoneal cavity. The uterine outline was well maintained (Figure 2B).

An appendectomy was performed, and generous peritoneal irrigation was carried out. Postoperatively, her lower abdominal pain subsided, and she was continued on intravenous antibiotics for seven days. She was discharged uneventfully on postoperative day 6 and was doing well at four weeks of follow-up.

**Figure 3:**
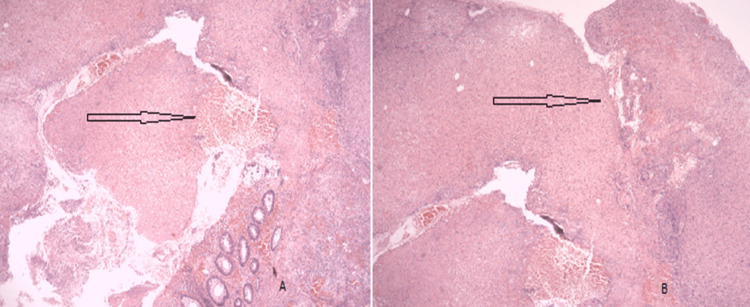
Histopathology image (A) Acute neutrophilic infiltration and fibrinoid deposits (arrow) in the appendix. (B) Breach in the continuity of the serosa of the appendix (arrow), diagnostic of appendicular perforation.

Histopathological examination revealed acute neutrophilic infiltration and fibrinoid deposits in the appendix (Figure 3A), along with a breach in the continuity of the serosa (Figure 3B), diagnostic of appendicular perforation. A final diagnosis of appendicular perforation, probably causing first-trimester abortion, was made.

## Discussion

Acute appendicitis complicates approximately one in 1000 pregnancies [4]. Pregnant women are less likely to develop appendicitis than age-matched, nonpregnant women [8]. During pregnancy, the incidence is slightly higher in the second trimester than in the first trimester or postpartum, and it is lowest in the third trimester [9]. Our patient initially presented in the first trimester, which is a rare occurrence.

Appendiceal perforation is reported in 13-20% of patients with acute appendicitis, with higher rates in men (18%) than in women (13%) [10]. Only about one in five patients with perforated appendicitis present within 24 hours of symptom onset [10]. In our case, the patient presented 48 hours after symptom onset. Delayed diagnosis in pregnant women with complicated appendicitis significantly increases the risk of maternal complications and fetal loss [4-6,11]. Each day of delay to surgery increases the odds of preterm delivery, preterm labor, or abortion by 23% [5]. Composite 30-day postoperative morbidity has been reported to be slightly higher in pregnant women compared with nonpregnant women (3.9% vs 3.1%), though not statistically significant, except for pneumonia, which was more frequent in pregnant women (0.7% vs 0.2%) [6]. A large retrospective study on maternal-fetal outcomes found that pregnant women with acute appendicitis were twice as likely to suffer fetal loss compared with those without appendicitis (absolute incidence, 0.2%), and they had a fivefold increased risk of inpatient maternal death [11].

Patients with uncomplicated appendicitis may be managed medically with antibiotics or expectant treatment, while acute perforated appendicitis generally requires emergency appendectomy, except in cases of periappendicular abscess [3]. In such patients, especially those with a longer duration of symptoms and phlegmon or abscess formation, immediate surgery carries higher morbidity due to dense adhesions and inflammation. Complications such as postoperative abscess or enterocutaneous fistula may arise, sometimes necessitating ileocolectomy or cecostomy.

Several scoring systems have been developed to standardize the diagnosis of acute appendicitis in nonpregnant populations, combining clinical and laboratory findings. These include the Appendicitis Inflammatory Response score, Alvarado score, Pediatric Appendicitis Score, and the Adult Appendicitis Score [7]. Recently, in 2023, the first nomogram for predicting complicated appendicitis in pregnancy was developed [4]. It incorporates three parameters: gestational age, CRP level with a cutoff of 34.82 mg/L, and neutrophil percentage ≥85.35%, with an area under the curve of 0.872 for predicting complicated appendicitis. In our case, both the CRP level (33.8 mg/L) and neutrophil percentage (79.1%) at initial presentation were below these cutoffs. This highlights that although biochemical parameters can aid diagnosis, they must be complemented with strong clinical suspicion and advanced imaging for timely detection.

The Swedish national guideline for the diagnosis and management of acute appendicitis in adults and children, published in 2025, also addresses considerations during pregnancy [12]. It recommends that for suspected appendicitis in pregnancy, blood tests including leukocytes, neutrophils, CRP, and a urine sample should always be obtained. Ultrasonography is advised as the first-line diagnostic modality, followed by MRI if available. CT scanning may be considered when complicated appendicitis is strongly suspected, particularly if ultrasound is inconclusive and MRI is unavailable or would delay diagnosis. In our case, a CT scan was performed after inconclusive ultrasonography, given the high cost of MRI and the urgency of diagnosis.

In cases of suspected appendicitis in pregnancy, observation with repeat blood tests and ultrasonography may be reasonable if the patient is not acutely unwell. Emergency appendectomy may be required, and diagnostic laparoscopy appears safe, particularly in early pregnancy [12]. However, evidence regarding risks and benefits remains limited and conflicting. In our case, laparoscopy was initially attempted but was converted to open laparotomy due to adhesions. The Swedish guidelines recommend a prophylactic dose of broad-spectrum antibiotics for appendectomy, postoperative antibiotics for complicated appendicitis, and no postoperative antibiotics for uncomplicated cases. A recent systematic review and meta-analysis comparing antibiotics versus surgery in pregnant women with acute appendicitis found that those managed with antibiotics had a lower risk of preterm labor but a higher risk of complications [13]. The authors concluded that the increased risk of complications warrants caution in considering antibiotics as first-line management during pregnancy.

Septic abortion can closely mimic acute complicated appendicitis. In both conditions, patients may present with systemic inflammatory response syndrome features such as fever, leukocytosis, neutrophilia, and elevated CRP. Differentiation requires careful history-taking, clinical examination, and imaging with ultrasound and/or MRI (preferred) or CT. A recent report described acute suppurative appendicitis with perforation in mid-pregnancy [14]. Our case was unique in that appendiceal perforation occurred in the first trimester, a rare event. The presentation mimicked septic abortion, given the history of recent surgical evacuation. A high index of suspicion, advanced imaging, and timely surgery enabled accurate diagnosis and optimal management. Unfortunately, the histopathological report of the evacuated products from the local hospital could not be retrieved. A pathology report showing no infection in the placental tissue would have ruled out a coincidental abortion from another cause. Thus, the causal relationship remains only probable.

## Conclusions

Acute appendicitis occurs in about one in 1000 pregnancies and may present as complicated or uncomplicated, depending on whether necrosis and/or perforation of the appendix is present. Delayed diagnosis and treatment of complicated appendicitis in pregnancy increase the risk of maternal complications and fetal loss. Our case was unusual, as the patient presented with appendiceal perforation in the first trimester, which is rare. The perforation likely led to inevitable abortion, necessitating suction evacuation, and mimicked septic abortion post-evacuation. A high index of suspicion, supported by advanced imaging, facilitated timely diagnosis and management. In the future, the use of pregnancy-specific nomograms may further aid early recognition of complicated appendicitis and guide optimal treatment.
